# Supplementary figures and images for: Metabolic and inflammatory axes of insulin resistance in normoglycemic adults with high HIV burden
Source: PLOS Glob Public Health. 2026 Jan 2;6(1):e0005589. doi: 10.1371/journal.pgph.0005589 (PMC12788187; doi:10.1371/journal.pgph.0005589)

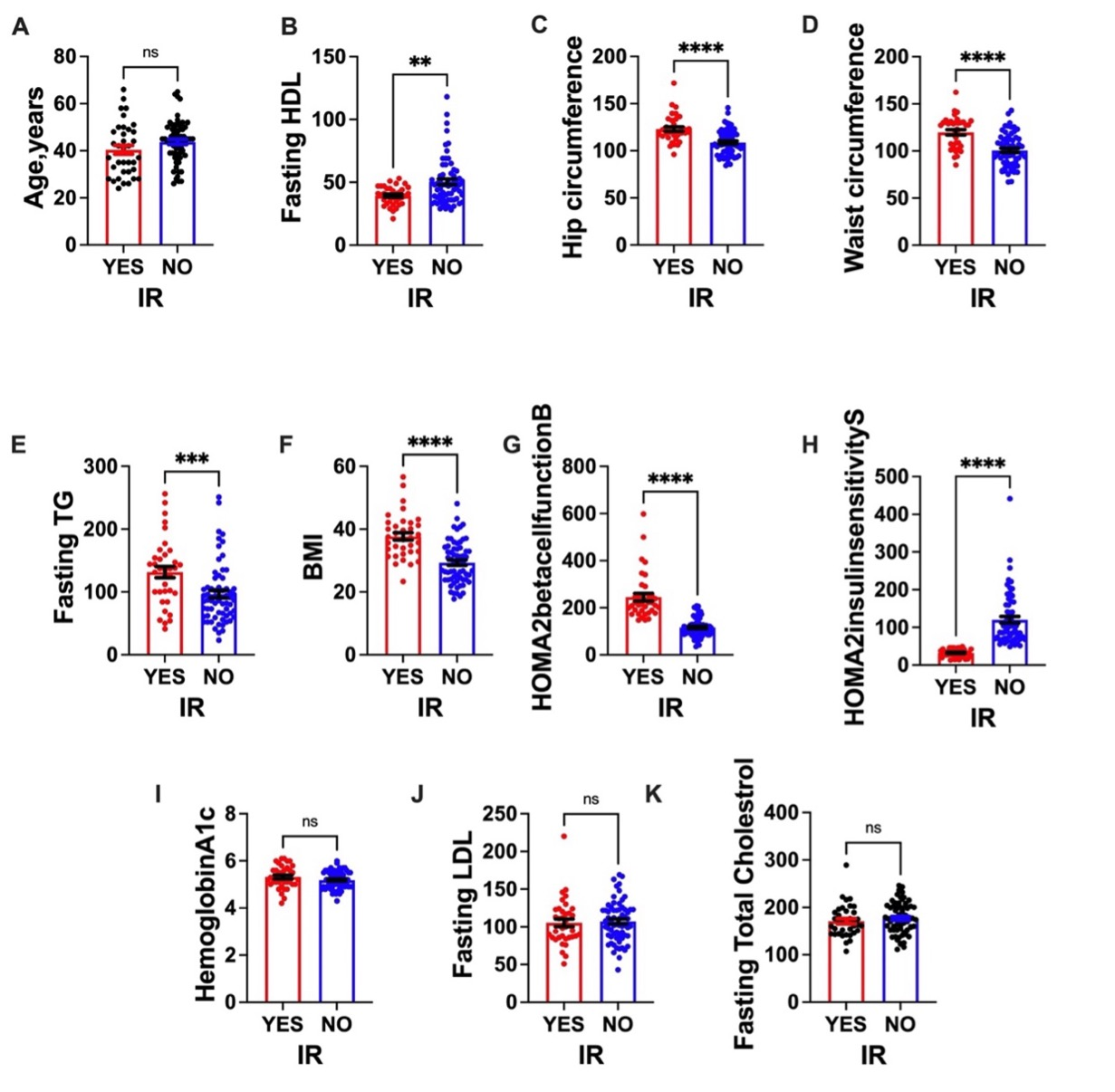

Supplement: S1 Fig — This figure shows the median (interquartile range, IQR) between people with and without IR, respectively: A) Age, 39 (31.00, 48.75) vs 44.5 (38.25, 49.00)/years, p = 0.074, B) Fasting HDL, 40 (33.50, 45.00) vs 47.5 (36.25, 56.75)/, mg/dL p = 0.0023, C) Hip circumference, 120.2(115.8, 131.1) vs 107.0(98.25, 119.2)/cm, p = < 0.0001 D) Waist circumference, 125.2 (104.0, 130.5) vs 98.8 (86.60, 113.4)/cm, p = < 0.0001, E) Fasting TG, 136 (98.50,157.8) vs 86.5 (62.00, 107.0)/, mg/dL p = 0.0005, F) BMI, 37.1 (32.85, 41.80) vs 29.03 (23.89, 33.98)/ kg/m², p = < 0.0001, G) HOMA2 beta cell function_B, 212.8 (181.0, 253.4) vs 109.2 (90.90, 133.0)/%B, p = < 0.0001, H) HOMA2 insulin sensitivity_S, 34.1 (23.38, 42.55) vs 99.6/%S, p = < 0.0001, I) HemoglobinA1c, 5.2 (5.000, 5.675) vs 5.6 (4.900, 5.500)/%, p = 0.155, J) Fasting LDL, 99 (86.00, 122.3) vs 105.5 (88.00, 123.0)/ mg/dL p = 0.545, K) Fasting Total Cholesterol, 165 (144.5, 191.3) vs 175.5 (152.0, 201.5)/ mg/dL, p = 0.290, IR, Insulin resistance, HDL; High-Density Lipoprotein, TG; Triglycerides, TNF-α; Tumour Necrosis Factor-alpha, HOMAIR; Homeostatic Model Assessment of Insulin Resistance, HOMA2; Homeostatic Model Assessment (version 2), IL5; Interleukin-5, IL6; Interleukin-6, IL10; Interleukin-10, IL7; Interleukin-7, C52; methylbutyryl, C5; Complement Component 5, C2uM; Acetylcarnitine, C2C3C5ratio; Acylcarnitine Ratio, C3uM; Propionylcarnitine. (TIF) [file pgph.0005589.s002.tif]

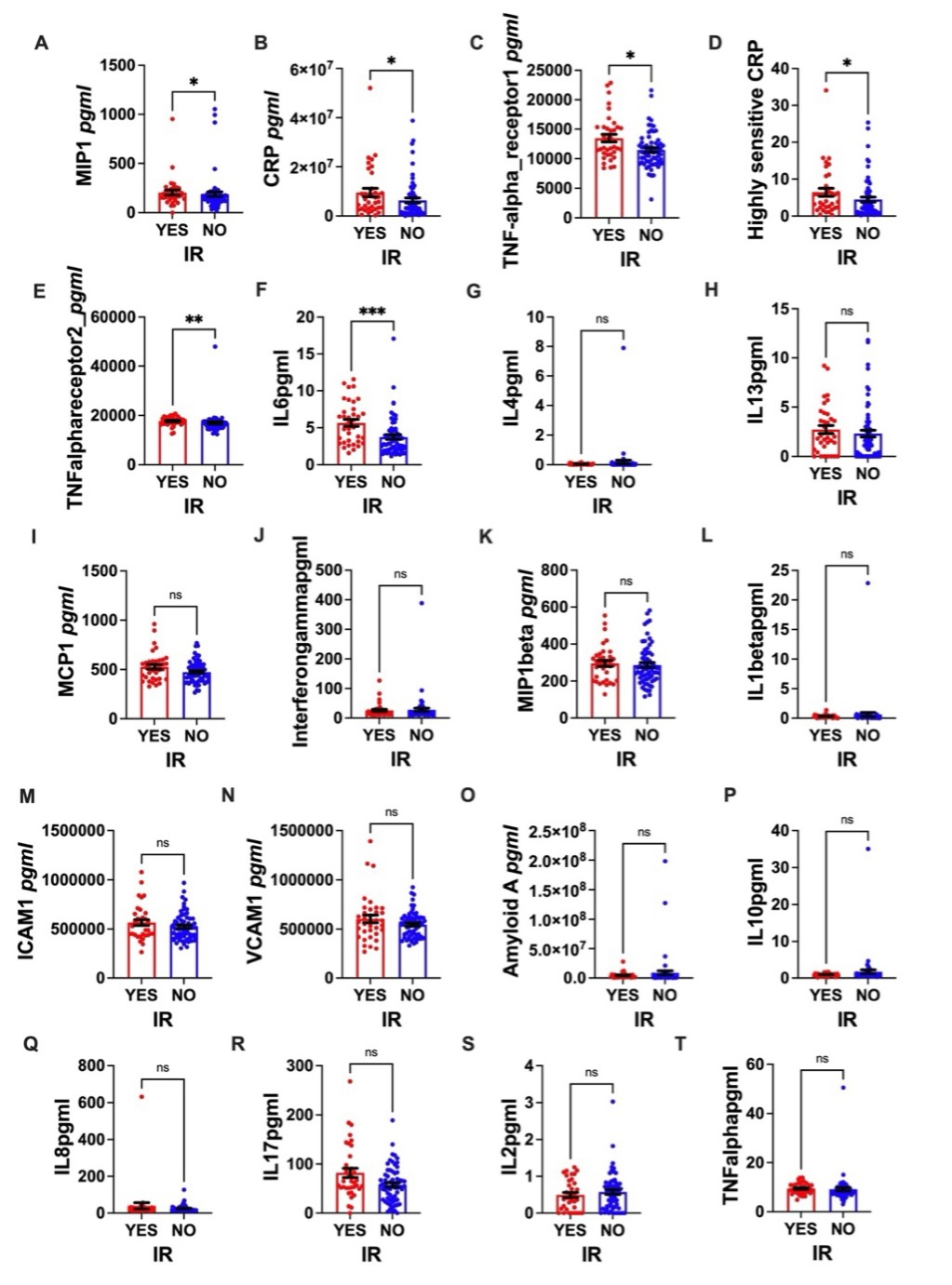

Supplement: S2 Fig — This figure shows the median (interquartile range, IQR) between people with and without IR, respectively: A) MIPalpha1, 180 (141.8, 237.3) vs 155 (114.3, 184.0)/ pgml, p = 0.039, B) CRP, 6275935 (2948899, 10699087) vs 3213787(1038752, 8591048)/ pgml p = 0.0204, C) TNFα receptor 1, 11913 (10759, 15334) vs 11165 (9394, 12710)/ pgml, p = 0.012, D) Highly sensitive CRP 4.800 (2.325, 7.700) vs 2.400 (0.8000,6.400)/ pgml, p = 0.018, E) TNFα receptor 2, 17832 (16582, 19215) vs 16664 (15399 17750)/, pgml p = 0.0016, F) IL6, 5.370 (3.020, 7.423) vs 2.900 (1.985, 4.628)/ pgml, p = < 0.0001, G) IL4, 0.024 (0.00525, 0.05475) vs 0.033 (0.000, 0.08175)/ pgml, p = 0.675, H) IL13, 2.050 (0.7750, 4.175) vs 1.450 (0.050, 2.950)/ pgml, p = 0.185, I) MCP1, 521.5 (407.3, 582.8) vs 451 (387.5, 542.0)/ pgml, p = 0.078, J) Interferon gamma, 18.2 (12.68, 28.13) vs 16.8 (12.95, 26.33)/ pgml, p = 0.712, K) MIP1beta, 298.5 (202.8, 336.0) vs 262 (211.5, 348.3)/ pgml, p = 0.445, L) IL1beta, 0.310 (0.1145, 0.445) vs 0.23 (0.125, 0.391)/ pgml, p = 0.596, M) ICAM1, 548417 (443116, 644533) vs 502401 (416088, 602580)/ pgml, p = 0.253, N) VCAM1, 579836 (458192, 693304) vs 552698 (427486, 616831)/ pgml, p = 0.301, O) Serum Amyloid A, 2573068 (1554338, 5714894) vs 1765767 (895165, 5971649)/ pgml, p = 0.153, P) IL10, 1.02 (0.745, 1.245) vs 0.94 (0.693, 1.518)/ pgml, p = 0.982, Q) IL8, 19.7 (14.3, 30.53) vs 17.65 (15.33, 26.35)/ pgml, p = 0.776, R) IL17, 63.6 (51.05, 111.4) vs 55 (27.15, 80.25)/ pgml, p = 0.052, S) IL2, 0.42 (0.1058, 0.8665) vs 0.59 (0.172, 0.787)/ pgml, p = 0.464, T) TNFalpha, 8.9 vs 8.2 (7.670, 10.880)/ pgml, p = 0.0565. IR, Insulin resistance, TNF-α; Tumour Necrosis Factor-alpha, IL5; Interleukin-5, IL6; Interleukin-6, IL10; Interleukin-10, IL7; Interleukin-7, C52; methylbutyryl, C5; Complement Component 5. (TIF) [file pgph.0005589.s003.tif]

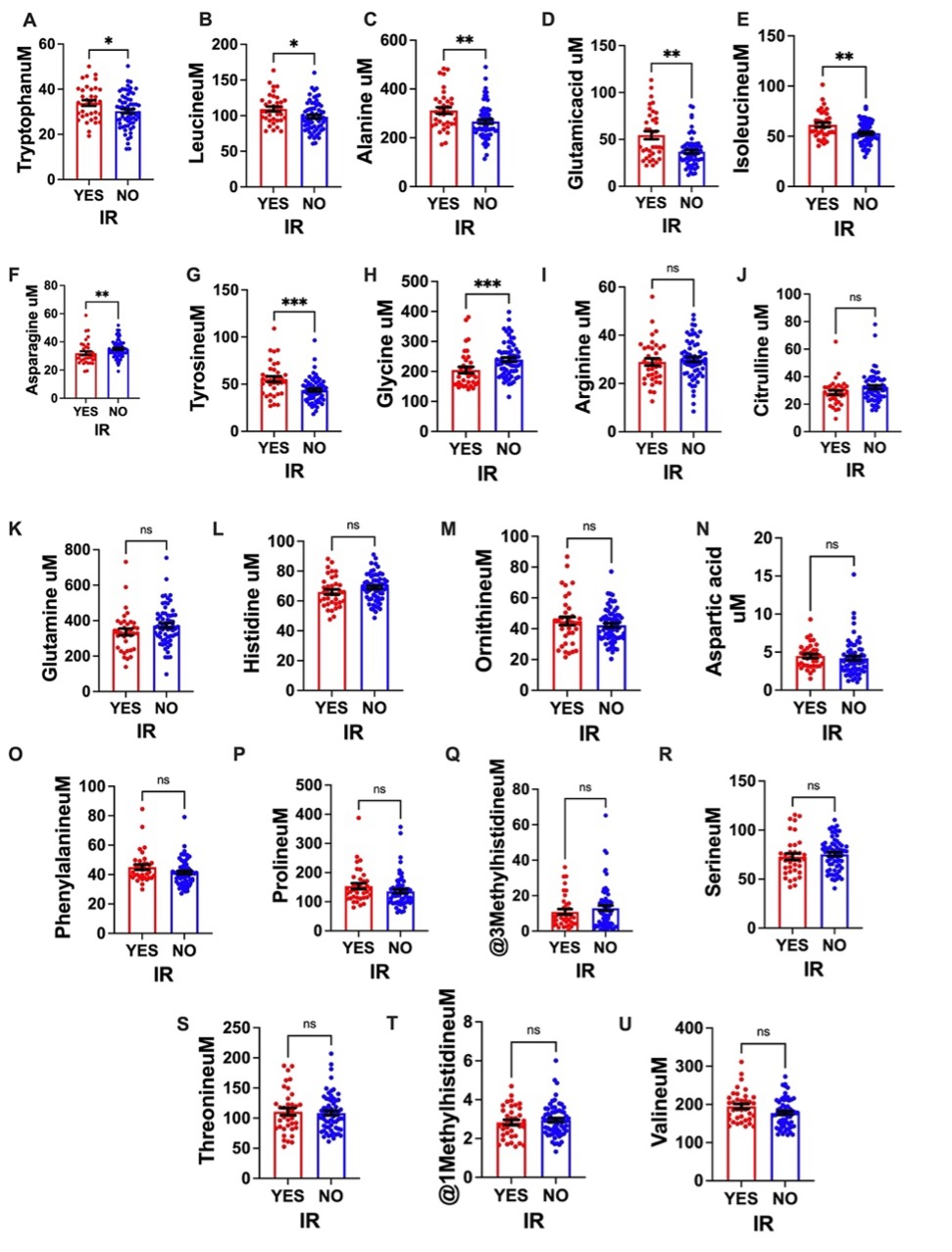

Supplement: S3 Fig — This figure shows the median (interquartile range, IQR) between people with and without IR, respectively: A) Tryptophan, 33.77 (28.17, 39.94) vs 30.13 (25.07, 35.51)/ uM, p = 0.034, B) Leucine, 106.3 (93.99, 121.5) vs 95.96 (82.96, 112.9)/ uM p = 0.013, C) Alanine, 302.4 (256.0356.5) vs 261 (216.0, 303.4)/ uM, p = 0.004, D) Glutamic acid 41.9 (33.19, 74.80) vs 35.4 (27.74, 43.47)/ uM, p = 0.001, E) Isoleucine, 59 (52.21, 71.31) vs (44.23, 62.13) 51.7/, uM p = 0.0041, F) Asparagine, 30.1 (26.92, 36.23) vs 34 (31.30, 38.70)/ uM, p = 0.0051, G) Tyrosine, 53.8 (40.17, 64.59) vs 42.9 (33.74, 51.18)/ uM, p = 0.0006, H) Glycine, 186.0 (156.9, 235.5) vs 231.3 (196.5, 268.0)/ uM, p = 0.0008, I) Arginine, 27.75 (22.14, 35.00) vs 29.61 (24.35, 35.49)/ uM, p = 0.393, J) Citrulline, 30.1 (23.30, 32.62) vs 34.1 (25.47, 37.61)/ uM, p = 0.0051, K) Glutamine, 336.9 (241.0, 386.9) vs 361.2 (307.4, 431.4)/ uM, p = 0.0719, L) Histidine, 64.9 (58.26, 72.50) vs 69.9 (61.76, 74.53)/ uM, p = 0.0995, M) Ornithine, 43.3 (31.58, 52.53) vs 41.2 (34.22, 48.80)/ uM, p = 0.6551, N) Aspartic acid, 4.5 (3.208, 5.466) vs 3.6 (2.615, 5.215)/ uM, p = 0.133, O) Phenylalanine, 42.5 (38.10, 47.82) vs 40.8 (34.80, 45.24)/ uM, p = 0.0766, P) Proline, 137.3 (113.8, 175.4) vs 126.4 (98.72, 152.1)/ uM, p = 0.0719, Q) @3Methylhistidine, 7.5 (4.139, 15.43) vs 9.5 (3.428, 16.97)/ uM, p = 0.6397, R) Serine, 69.9 (57.96, 82.86) vs 77.6 (61.77, 85.89)/ uM, p = 0.275, S) Threonine, 106.9 (83.33, 130.3) vs 104.5 (84.21, 129.5)/ uM, p = 0.8498, T) @1Methylhistidine, 2.73 (2.143, 3.528) vs 2.92 (2.375, 3.412)/ uM, p = 0.561, U) Valine, 186.8 (161.5, 218.0) vs 174.2 (147.2, 209.4)/ uM, p = 0.0602. IR, Insulin resistance. (TIF) [file pgph.0005589.s004.tif]

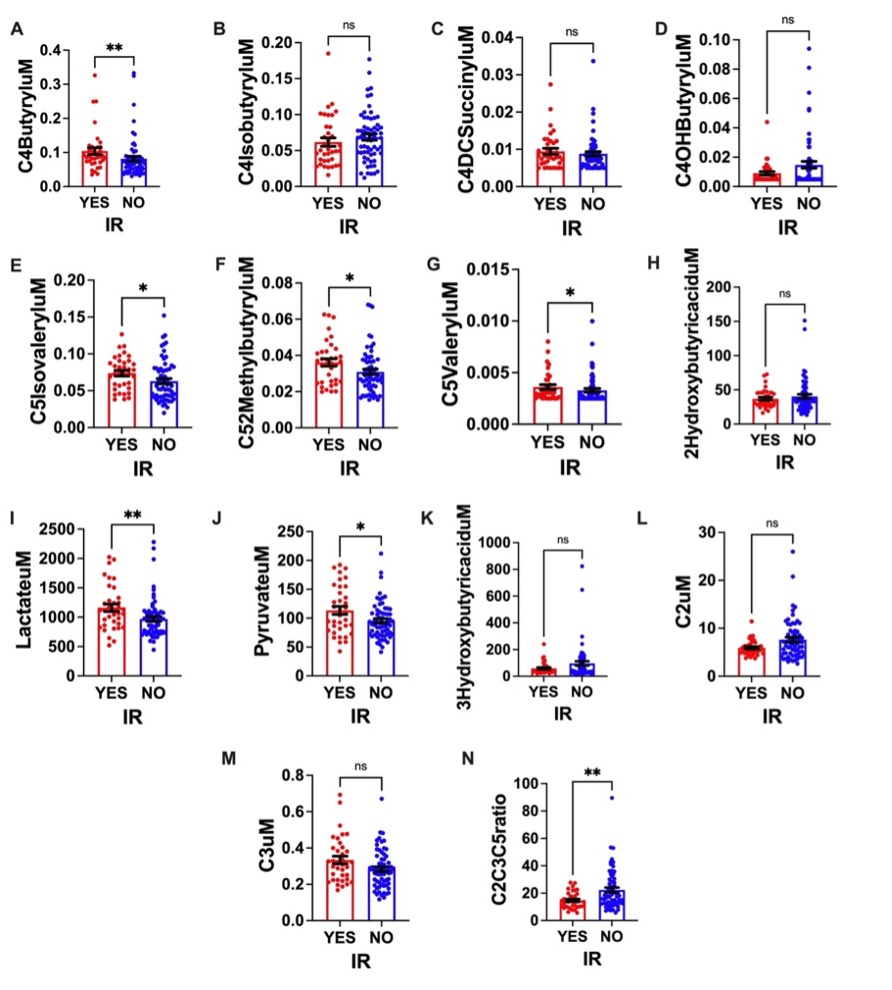

Supplement: S4 Fig — This figure shows the median (interquartile range, IQR) between people with and without IR, respectively: A) C4Butyryl, 0.088 (0.0718, 0.1161) vs 0.069 (0.0461, 0.083)/ uM, p = 0.0019, B) C4Isobutyryl, 0.0498 (0.036, 0.083) vs 0.068 (0.046, 0.085)/ uM p = 0.2687, C) C4DCSuccinyl, 0.007995 (0.006, 0.012) vs 0.00768 (0.006, 0.0099)/ uM, p = 0.472, D) C4OHButyryl 0.0055 (0.005, 0.012) vs 0.005 (0.005, 0.017)/ uM, p = 0.419, E) C5Isovaleryl, 0.0736 (0.054, 0.0899) vs 0.0553 (0.042, 0.0796)/, uM p = 0.0118, F) C52Methylbutyryl, 0.036 (0.0252, 0.0421) vs 0.028 (0.023, 0.0371)/ uM, p = 0.0305, G) C5Valeryl, 0.0030 (0.00305, 0.0043) vs 0.0025 (0.0025, 0.0035)/ uM, p = 0.028, H) 2Hydroxybutyric acid, 31.9 (28.14, 45.74) vs 34.0 (24.34, 48.06)/ uM, p = 0.997, I) Lactate, 1112 (867.5, 1317) vs 919.5 (739.8, 1076)/ uM, p = 0.0028, J) Pyruvate, 106.8 (79.55, 152.0) vs 90.5 (72.03, 110.0)/ uM, p = 0.0405, K) 3Hydroxybutyricacid, 41.16 (31.33, 70.85) vs 53.99 (31.00, 131.5)/ uM, p = 0.301, L) C2, 5.64 (4.917, 6.665) vs 6.79 (4.588, 9.435)/ uM, p = 0.097, M) C3, 0.3113 (0.2246, 0.4025) vs 0.2773 (0.1983, 0.3499)/ uM, p = 0.0518, N) C2C3C5ratio, 13.83 (10.18, 19.11) vs 17.86 (12.51, 29.56)/ uM, p = 0.0045, IR, Insulin resistance, C52; methylbutyryl, C2uM; Acetylcarnitine, C2C3C5ratio; Acylcarnitine Ratio, C3uM; Propionylcarnitine. (TIF) [file pgph.0005589.s005.tif]

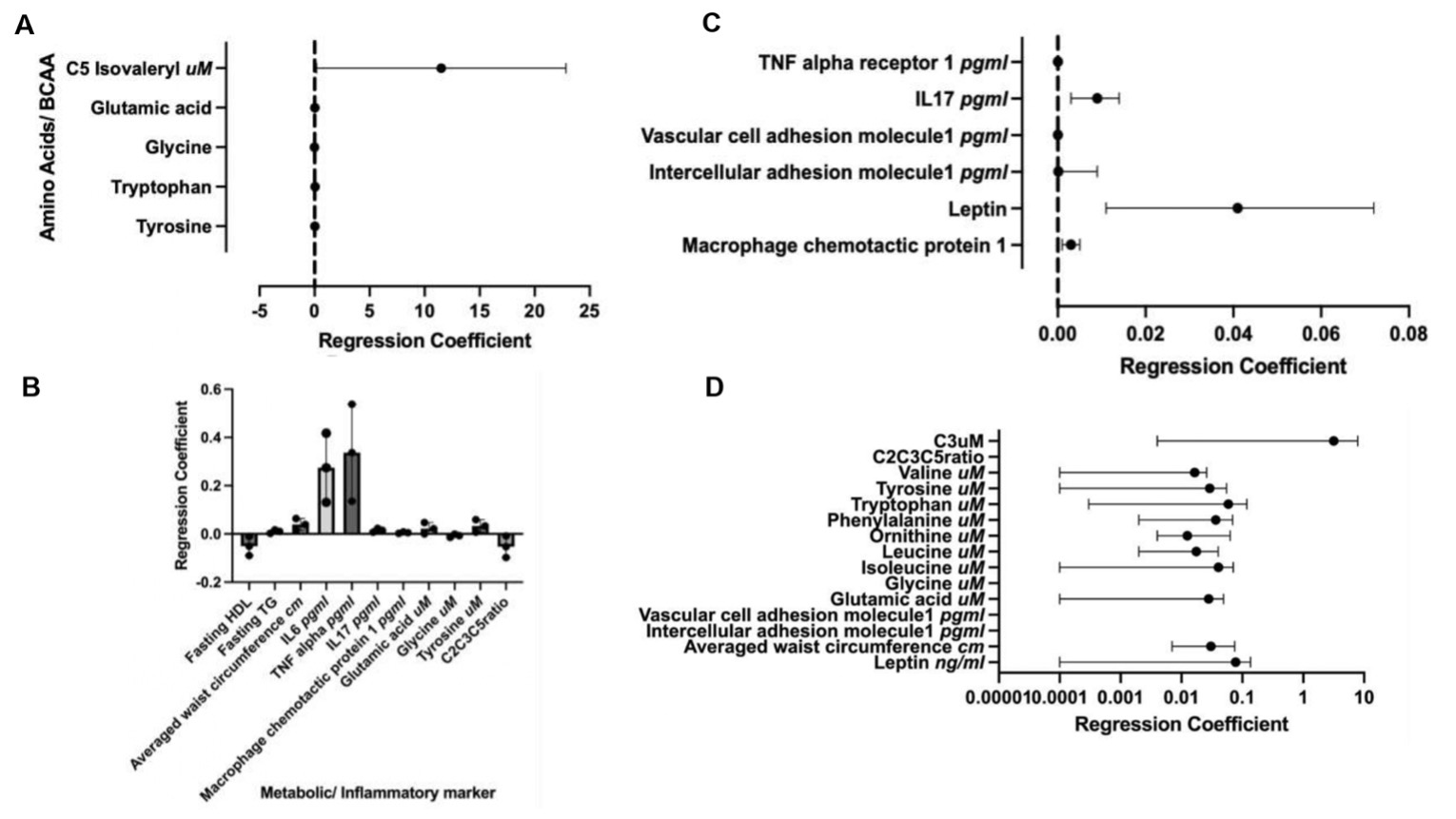

Supplement: S5 Fig — Panel A presents a forest plot showing the overall associations of key metabolites, including isovaleryl (C5), glutamic acid, glycine, tryptophan, and tyrosine, with IR, adjusted for age, sex, and BMI. Panel B highlights markers significantly associated with IR in individuals with obesity, including IL-6, fasting HDL, glutamic acid, glycine, IL-5, TNF-α, IL-17, leptin, and MCP-1, adjusted for age and sex. Panel C depicts inflammatory markers—TNF-α1, IL-17, VCAM-1, ICAM-1, leptin, and MCP-1, associated with IR in the overall sample, adjusted for age, sex, and BMI. Panel D shows the corresponding associations in non-obese individuals, where C3uM, leptin, averaged hip circumference, tyrosine, tryptophan, and glycine were significantly linked to IR, adjusted for age and sex. (TIF) [file pgph.0005589.s006.tif]
