# Supplementary material for: Metabolic and inflammatory axes of insulin resistance in normoglycemic adults with high HIV burden
Source: PLOS Glob Public Health. 2026 Jan 2;6(1):e0005589. doi: 10.1371/journal.pgph.0005589 (PMC12788187; doi:10.1371/journal.pgph.0005589)
Supplement: Attachment — Submitted filename: Response to Reviewers.docx [file pgph.0005589.s008.docx]

**Response to Review Comments to the Author**

We thank the reviewer for their valuable comments that has improved our manuscript. Thank you for taking you time and dedicating it to help sharpen our scientific communication. We have now addressed all comments detailed below and hope that you are satified with this improved version of the manuscript.

**Reviewer #1:**

**Comment**

Title: The paper could benefit from a simpler title.

***Response:*** *New title: Metabolic and Inflammatory Axes of Insulin Resistance in Normoglycemic Adults*

**Comment**

Line 96: The authors stated that HOMA-IR is well validated and offer Ref# 4 (where they studied Chinese participants) as proof. However, there is a body of literature which disputes this. Please see BMC Res Notes. 2014;7:98. doi: 10.1186/1756-0500-7-98 and Diabetes Care. 2013, 36 (4): 845-853. 10.2337/dc12-0840. They need to discuss this as a limitation.
***Response:***
*(Done, statement included in limitations sections)*

**Comment**

The paragraph starting on line 124 seems better placed before line 114, and lines 132-135 seem redundant.

***Response:***
*Redundancies reduced*. Thank you

**Comment**

Line 172: “Adipokines (leptin, adiponectin) and inflammatory markers were measured.” Please give the assays coefficients of variation (CVs) or sensitivities. This would also apply to the organic acids.
***Author Response:***

*We appreciate the reviewer’s request for detailed analytical performance characteristics. As this was a retrospective analysis utilizing previously generated laboratory data, the original dataset did not include full assay performance metrics such as coefficients of variation (CVs) or lower limits of detection for the adipokines, inflammatory markers, and organic acid panels. The assays were performed in accredited clinical and research laboratories adhering to internal and external quality-control procedures; however, the archived dataset did not retain the granular validation parameters required to report CVs for each analyte.*

**Comment**

Line 175: HOMA indices were calculated by the simple equations. However, the Oxford group has generally recommended using their online calculator to derive model-derived estimates rather than linear approximations. But then in line 202 the authors said they used the calculator. So, please clarify what was actually done. Finally, Lines 182-5 are redundant given they are describing what was said before in Line 175.

***Response:*** *we have now made the Clarification in the manuscript: HOMA2 IR was calculated using an online calculator. We have now removed the redundant lines.*

**Comment**

Line 228: The multivariable models are not described well enough for easy understanding on how the models were derived/created and the results generated. Could this be reworded?
***Response:*** *Multivariable regression analysis stratified by obesity status was removed from the study. The statement has since been removed.*

**Comment**

Line 240: Please define how you made a continuous variable, HOMA-IR, into a dichotomous variable. Were you using the upper quintile of the distribution or some other cutoff? Please justify why the cutoffs were chosen.
***Response:*** *Done. HOMA2-IR, initially calculated as a continuous quantitative measure of insulin resistance, was transformed into a dichotomous variable to classify participants into “insulin resistant” versus “non–insulin resistant” groups. To accomplish this, we applied a predefined threshold of HOMA2-IR 1.9, which is consistent with published epidemiological and metabolic research identifying early insulin resistance. This value as indicative of clinically meaningful insulin resistance. Values above 1.9 cutoff were coded as “1” (elevated insulin resistance), and values at or below the cutoff were coded as “0”*

**Comment**

Line 244: “Significant difference was also observed based on HIV status…” But these participants were all on HAART medications with viral suppression. So, HIV status may be a marker of drug exposure and the drugs were exerting metabolic effects.
***Author Response:***

*We thank the reviewer for this important clarification. The sentence in the Results section was intended solely as a descriptive summary of the observed statistical differences presented in the table, without inferring causality or mechanistic interpretation. We agree that in virally suppressed individuals on long-term HAART, “HIV status” may indeed reflect cumulative antiretroviral drug exposure rather than active viral effects. To avoid misinterpretation, we have now clarified this in the Discussion section, where we address the potential metabolic influence of HAART regimens and acknowledge that the observed differences may be attributable to antiretroviral therapy rather than HIV infection per se.*


**Comment**

Table 2 showed be labelled with the variables used in the model, i.e. age, sex, BMI. Also, would not waist be a better variable to adjust for fat mass, since it is more specific for visceral and ectopic fat than BMI?
***Response:*** *We thank the reviewer for this thoughtful observation. We agree that waist circumference is a more direct proxy for visceral adiposity and may, in some contexts, provide greater specificity than BMI for estimating central fat accumulation. However, in the present analysis, we elected not to include waist circumference in the multivariable model for two key reasons.*

*First, the study was not stratified by obesity status, and including both BMI and waist circumference simultaneously introduces a high degree of collinearity, as both variables capture overlapping components of general and central adiposity. Given our modelling objective, to adjust for overall body size while avoiding multicollinearity, we selected BMI as the primary anthropometric covariate.*

*Second, because waist circumference is strongly correlated with HOMA2-IR and may itself lie along the causal pathway between adiposity and insulin resistance, adjusting for it could lead to overadjustment bias. To maintain a parsimonious model and avoid obscuring true associations, we therefore opted to retain age, sex, and BMI as the adjustment variables.*

**Comment**

Figures: There is an overly large amount figures. Some of these could be summarized in the text or as supplementary figures.
***Author Response:***

*We thank the reviewer for this helpful observation. We agree that the initial submission contained a large number of figures, some of which presented descriptive patterns that can be effectively conveyed in text or placed in supplementary materials. To improve clarity, streamline the presentation, and align with journal expectations, we have now reduced the number of figures in the main manuscript. Specifically, figures that provide supportive but non-essential visual detail have been moved to the Supplementary Materials, while others have been summarized directly in the Results narrative.*

**Comment**

Line 507: Please discuss that reverse causality could also be an explanation of the associations as IR would directly lead to increased gluconeogenesis supported by protein turnover.
***Response:***
*Done. Statement discussing reverse causality associating IR, included.*

**THANK YOU AGAIN FOR YOUR REOMMENDATIONS AND SUGEGSTIONS**

**Reviewer #2:**

General review comments to the authours
The manuscript titled “Metabolic and Inflammatory Axes of Insulin Resistance in Normoglycemic Adults from the Observational Cohort Study of Adipose Tissue and Immune Activation: Adipocytokines, Lipids and Amino Acids Pathways” (PGPH-D-25-02739) presents a rigorous, well-organized, and ethically conducted investigation into the multifactorial mechanisms of insulin resistance (IR) in normoglycemic adults. The integration of metabolic, inflammatory, and adipokine markers represents a valuable contribution to metabolic and public health research.

1. Scientific and technical merit
The study is well-conceived and addresses a significant knowledge gap on early metabolic dysfunction before the onset of diabetes. The manuscript demonstrates methodological soundness and aligns with PLOS Global Public Health’s focus on translational and population-relevant research.

**Response: Thank you for highlighting our research and for providing complimentary and favourable feedback**

Comment

L43–L83 (Abstract): The abstract clearly presents the study background, objectives, methods, and conclusions. However, the prevalence of IR (L62–L63) is repeated and can be stated once for conciseness. The concluding sentence (L75–L79) effectively highlights the emergence of two IR phenotypes and should remain as the key takeaway.
***Response:***
*Done. Repetition of prevalence removed in the abstract section*

Comment

L87–L135 (Introduction): The rationale and context are strong, but the first two paragraphs (L87–L99) can be condensed to avoid redundancy regarding the roles of adipokines and cytokines. The study hypothesis (L114–L121) is clear and logically follows the background provided.
***Response:***
*We appreciate the reviewer’s feedback. The last two paragraphs of the introduction were condensed to reduce redundancy regarding the roles of adipokines and cytokines.*

*Overlapping sentences were removed or merged to streamline the narrative, thereby strengthening the overall clarity and focus of the introduction while preserving essential context for the study hypothesis.*

Comment

L124–L135: The mention of methodological variability in insulin resistance assessment is appropriate; however, citing comparative studies on HOMA2-IR validation would further strengthen this point.
Overall, the study presents a clear rationale and scientifically valid objectives that are appropriately addressed by the chosen methods.
***Response:*** *Citations have been added to strengthen and validate the submission*. Thank you for the positive feedback

2. Statistical analysis
The statistical methods are appropriate and executed rigorously, supporting the validity of the conclusions drawn.

Response: Thank you for the positive feedback


L211–L231 (Data Analysis Plan): The statistical workflow, including the use of Shapiro–Wilk test, Chi-square, Fisher’s exact, Pearson’s, and Spearman’s correlations, is well justified.

Response: Thank you for the positive feedback

Comment

The multivariable linear regression model (L225–L230) is correctly structured, but it would be helpful to state whether covariates were entered simultaneously or through stepwise selection.
***Response:*** *Thank you for the comment. For the multivariable linear regression analyses, covariates were selected a priori based on their established biological and clinical relevance to insulin resistance, and all prespecified covariates were entered into the model in a stepwise selection.*

Comment

L365–L440 (Regression Analysis): Table 2 effectively presents β-coefficients and confidence intervals. Ensure consistency between univariate and multivariable models and verify that all adjustments (age, sex, BMI) were retained across analyses. Reporting adjusted R² values for transparency would strengthen interpretation.

***Response:*** *Reporting adjusted R² values have now been made to table 2. Thank you for this observation.*

Comment

L443–L459 (Figure 9): The forest plot is an effective visual summary. Confirm that all variables plotted correspond to those in Table 2, and that abbreviations (e.g., C3uM, IL-17, MCP-1) are fully defined in the legend.
The statistical evidence adequately supports the identification of two mechanistically distinct IR phenotypes.

***Response:***

*Thank you for the observation. Not all variables from Table 2 were included in the forest plot. The forest plot displays only the primary exposure variables, whereas the following covariates were excluded from the plot: baseline glucose, baseline insulin (µU/mL), 90-minute glucose, 90-minute insulin (µU/mL), 120-minute glucose, baseline HOMA-IR, HOMA2 insulin sensitivity (S), and HOMA2 β-cell function (B). We have also confirmed that all abbreviations for the variables plotted (C3uM, IL-17, MCP-1) are fully defined in the figure legend.*

Comment

3. Ethical and data considerations
L137–L145 (Ethics Statement): Ethical approval (IRB# 121175) and informed consent are appropriately documented. The authors followed the principles of the Declaration of Helsinki and ensured participant confidentiality, fulfilling PLOS ethical standards.
Response: Thank you for the positive feedback

Data Availability Statement (page 3): The authors indicate that data are available upon request due to privacy and ethical restrictions. While acceptable for sensitive datasets, this approach is partly compliant with PLOS’s open-data policy. The authors should deposit a de-identified dataset or summary-level data in a public repository (e.g., Zenodo or Dryad) and include the repository DOI in the final version.

***Response:***
*We appreciate the reviewer’s comment. De-identified summary-level data supporting the findings of this study are subject to institutional and ethical restrictions; therefore, deposition into a public repository requires prior approval from our Research Ethics Committee. We have initiated the process to obtain this approval and will deposit the de-identified dataset or summary-level data in an appropriate public repository (e.g., Zenodo or Dryad) during the production stage, once clearance is granted.*

*In the interim, the data remain available from the corresponding author upon reasonable request, in accordance with ethical and privacy requirements.*

Comment

4. Clarity and presentation
The manuscript is written in clear, standard academic English, with strong organization and logical flow between sections.

Response: Thank you for the positive feedback

Comment

L239–L247 (Results – Participant Characteristics): The data are well described but could be presented more succinctly; the same statistics appear in both text and Table 1. Retain the values in the table and summarize patterns in the text.
***Response:*** *We thank the reviewer for this helpful suggestion. We agree that the initial description contained unnecessary repetition of numerical values already provided in Table 1. In response, we have revised this section to present a more concise, pattern-focused summary while retaining all detailed statistics in Table 1. The updated text now highlights key trends and relationships without duplicating the table content, thereby improving clarity and readability of the Results section.*

Comment

L281–L285 (Inflammatory Profile): The findings are clearly reported; ensure consistent presentation of units (pg/mL, µM) throughout.
***Response:*** *We thank the reviewer for this helpful observation. The units for all inflammatory and metabolic biomarkers are correctly and consistently presented in the corresponding tables. In the Results narrative (L281–L285), units were intentionally omitted to avoid redundancy, as this section serves only to summarize the patterns shown in the table rather than restate the full numeric values.*

Comment

L311–L316 (Amino Acids): The narrative is clear and well-supported by Figure 3.

Response: Thank you for the positive feedback

L463–L486 (Discussion – Main Findings): The summary of key findings is concise and well aligned with the data.

Response: Thank you for the positive feedback

L501–L523 (Discussion – Mechanistic Pathways): This section is detailed and scientifically sound but could be reduced slightly to improve readability.
***Response:***

*We thank the reviewer for this helpful suggestion. In response, we have streamlined this section by removing redundant statements, consolidating overlapping concepts, and improving transitions between mechanistic pathways. The revised paragraph maintains all essential scientific content while presenting the key mechanistic insights in a more succinct and readable format.*

Comment

L529–L553 (Conclusion): The conclusion appropriately synthesizes the study’s implications. Consider linking the findings to practical applications in early diabetes screening or metabolic risk assessment.
***Response:***

*Thank you for this valuable suggestion. We have revised the conclusion to more explicitly link our findings to potential practical applications in early diabetes screening and metabolic risk assessment.*

Minor editorial points:
Maintain consistency in abbreviation definitions (IL-6, TNF-α, HOMA2-IR).
***Response: Done***

Check uniformity of units (mg/dL vs. mmol/L) and ensure SI compliance.
***Response: Done***
Correct minor typographical issues (spacing, punctuation in tables).
***Response: Done***

5. Contribution and relevance
The study advances understanding of early insulin resistance and distinguishes between metabolic and inflammatory-lipotoxic pathways. The identification of glycine as inversely associated with IR and propionylcarnitine (C3uM) as a metabolic biomarker offers translational potential. These findings have important public health implications, suggesting early biomarkers for metabolic risk among normoglycemic individuals, especially in populations with high HIV prevalence.

Response: Thank you for the positive feedback

6. Summary recommendations
The manuscript is technically sound, ethically compliant, and clearly written. The conclusions are well supported by the data. With minor editorial refinements and compliance with open-data requirements, this study will make a strong contribution to the literature on metabolic and inflammatory determinants of insulin resistance.

Response: Thank you for the positive feedback

Required Minor Revisions:
• Condense repetitive numerical reporting in the Results section (L239–L247, L281–L285).
***Response: Done***
• Add details on the variable entry approach in regression modelling (L225–L230).
***Response: Done***
• Standardize units and abbreviations throughout.
***Response: Done***
• Deposit de-identified data or summary tables in a public repository and update the Data Availability Statement.
***Response: Done***
• Reduce background repetition in the Introduction (L87–L99) and Discussion (L501–L523).
***Response: Done***
