# Supplementary material for: Metabolic and inflammatory axes of insulin resistance in normoglycemic adults with high HIV burden
Source: PLOS Glob Public Health. 2026 Jan 2;6(1):e0005589. doi: 10.1371/journal.pgph.0005589 (PMC12788187; doi:10.1371/journal.pgph.0005589)
Supplement: Attachment — Submitted filename: Response to Reviewer Comments (1).docx [file pgph.0005589.s009.docx]

**Response to Reviewer Comments**

We sincerely thank the Academic Editor for the insightful and constructive comments. We have carefully revised the manuscript to address the concern regarding the specificity of our study population and the need to clearly emphasize the high HIV burden among participants.

**1. Title Revision**

As recommended, the title has been updated to explicitly reflect the population under study:

**Revised Title:**
*Metabolic and Inflammatory Axes of Insulin Resistance in Normoglycemic Adults with High HIV Burden*

**2. Incorporation of HIV Context Throughout the Manuscript**

We have strengthened the manuscript by explicitly acknowledging the high prevalence of HIV within the study population and clarifying how HIV infection and antiretroviral therapy (ART) may influence metabolic and inflammatory pathways relevant to insulin resistance. These additions have been incorporated into the:

- **Discussion Section**
  – Multiple passages have been revised to emphasize that the metabolic and inflammatory signatures observed may be shaped by HIV-related immune activation, chronic inflammation, and/ or ART exposure.
  – Comparisons with other studies now explicitly highlight the biological uniqueness of PLWH and caution against assuming applicability to HIV-uninfected populations.
- **Conclusion Section**
  – The conclusion has been rewritten to reinforce that the findings arise from a high–HIV-burden cohort and may not generalize to normoglycemic individuals without HIV infection.
  – Mechanistic interpretations are now contextualized within the immune-metabolic milieu of PLWH.
- **Limitations Section**
  – The limitations have been expanded to note that the predominance of PLWH limits external generalizability, and future studies are needed in HIV-uninfected cohorts.

**3. Ensuring Accurate Interpretation of Findings**

In accordance with the reviewer’s request, we have revised the language throughout the manuscript to ensure that readers do not mistakenly interpret the findings as universally generalizable. We now consistently articulate that observed associations may be modified, amplified, or confounded by factors unique to HIV infection or ART exposure.

**4. Figures** have been tested in the PLOS’s NAAS tool and ‘passed’
